# A randomized trial to compare exercise treatment methods for patients after total knee replacement: protocol paper

**DOI:** 10.1186/s12891-015-0761-5

**Published:** 2015-10-16

**Authors:** Sara R. Piva, Charity G. Moore, Michael Schneider, Alexandra B. Gil, Gustavo J. Almeida, James J. Irrgang

**Affiliations:** Department of Physical Therapy, University of Pittsburgh, Bridgeside Point 1, 100 Technology Drive, Suite 210, Pittsburgh, PA 15219-3130 USA; Research in Biostatistics, Dickson Advanced Analytics, Carolinas HealthCare System, Charlotte, North Carolina USA; Department of Orthopaedic Surgery, University of Pittsburgh School of Medicine, Pittsburgh, Pennsylvania USA

**Keywords:** Knee osteoarthritis, Total knee arthroplasty, Total knee replacement, Rehabilitation, Exercise program, Physical therapy

## Abstract

**Background:**

Although the outcome of total knee replacement (TKR) is favorable, surgery alone fails to resolve the functional limitations and physical inactivity that existed prior to surgery. Exercise is likely the only intervention capable of improving these persistent limitations, but exercises have to be performed with intensity sufficient to promote significant changes, at levels that cannot be tolerated until later stages post TKR. The current evidence is limited regarding the effectiveness of exercise at a later stage post TKR. To that end, this study aims to compare the outcomes of physical function and physical activity between 3 treatment groups: clinic-based individual outpatient rehabilitative exercise during 12 weeks, community-based group exercise classes during 12 weeks, and usual medical care (wait-listed control group). The secondary aim is to identify baseline predictors of functional recovery for the exercise groups.

**Methods/Design:**

This protocol paper describes a comparative effectiveness study, designed as a 3-group single-blind randomized clinical trial. Two hundred and forty older adults who underwent TKR at least 2 months prior will be randomized into one of the three treatment approaches. Data will be collected at baseline, 3 months, and 6 months. The wait-listed control group will be randomized to one of the 2 exercise groups after 6 months of study participation, and will complete a 9-month follow-up. Primary outcome is physical function measured by the Western Ontario and McMaster Universities Osteoarthritis Index Physical Function Subscale (WOMAC-PF). Physical function is also measured by performance-based tests. Secondary outcomes include performance-based tests and physical activity assessed by a patient-reported survey and accelerometry-based physical activity monitors. Exploratory outcomes include adherence, co-interventions, attrition, and adverse events including number of falls. Linear mixed models will be fitted to compare the changes in outcome across groups. Logistic regression will identify patient characteristics that predict functional recovery in the exercise groups. Instrumental variable methods will be used to estimate the efficacy of the interventions in the presence of non-compliance.

**Discussion:**

Results will inform recommendations on exercise programs to improve physical function and activity for patients at the later stage post TKR and help tailor interventions according with patients’ characteristics.

**Trial registration:**

ClinicalTrials.gov Identifier NCT02237911.

## Background

Over 4 million U.S. adults currently live with a total knee replacement (TKR) and it is projected that greater than 3 million TKRs will be performed annually by 2030 [1]. By most metrics, TKRs are successful surgeries, as they reduce pain, improve quality of life, and are cost-effective [2, 3]. However, long-term functional and activity limitations due to the chronic joint disease prior to surgery do not spontaneously resolve after TKR [3–9]. A study found that after one year, 52 % of subjects who had received TKR surgeries continued to have substantial limitations during activities such as kneeling, squatting, turning and cutting, carrying loads, tennis, dancing, gardening, and sexual activity, in contrast to only 22 % of matched controls [10]. Subjects post-TKR are also at increased risk for falls [11] and do not reach recommended levels of physical activity to prevent morbidity [12].

Exercise therapy is a simple solution to alleviate these persistent functional limitations and enhance TKR outcome. However, studies demonstrated only modest benefits of exercise programs during the first couple of months (early stage) post-TKR [13]. These modest benefits are not surprising as exercise programs in the early post-operative stage (when patients are still healing from the TKR) after surgery are mostly focused on improving simple knee movement and independent mobility. Participation in more comprehensive exercise programs that target the persistent muscle weakness, deconditioning, and functional limitation is likely the only way to reverse the substantial deficits that existed for years or decades before surgery and persist after TKR. To accomplish these more comprehensive goals, exercises have to be performed with an intensity sufficient to promote important strength and functional changes, at levels that cannot be tolerated until later stages (after 2 months) post TKR.

Emerging evidence indicates that intense exercise at later stages post-TKR is safe and promotes substantial functional recovery [14–17]. However, the evidence is restricted to a few small studies of explanatory design weakened by methodological limitations, which leaves patients and providers without guidance on recommendations to maximize the benefits of TKR. While the current usual care typically consists of premature discharge from rehabilitation before exercises can be intensified to enhance functional outcome, an increasing number of patients are seeking additional outpatient rehab or community-based exercise. While these services are readily available, there is a lack of evidence about their benefits and harms. Also, these services are infrequently covered by health insurance, which adds substantial financial burden and barriers to compliance.

To that end, we propose a pragmatic study to investigate the effectiveness of exercise programs during the later stage (>2 months) post-TKR rehabilitation. The primary aim is to compare the outcomes of physical function and physical activity between 3 treatment groups: (1) clinic-based individual outpatient rehabilitative exercise, (2) community-based group exercise classes, and (3) usual medical care. The secondary aim is to identify baseline predictors of functional recovery for both exercise groups. The exploratory aim is to compare attrition, adherence, adverse events and co-interventions across treatment groups. Results of this study will inform the choice of interventions for the later stages after TKR and provide evidence to tailor interventions according with patient characteristics.

## Methods/Design

**Fig. 1:**
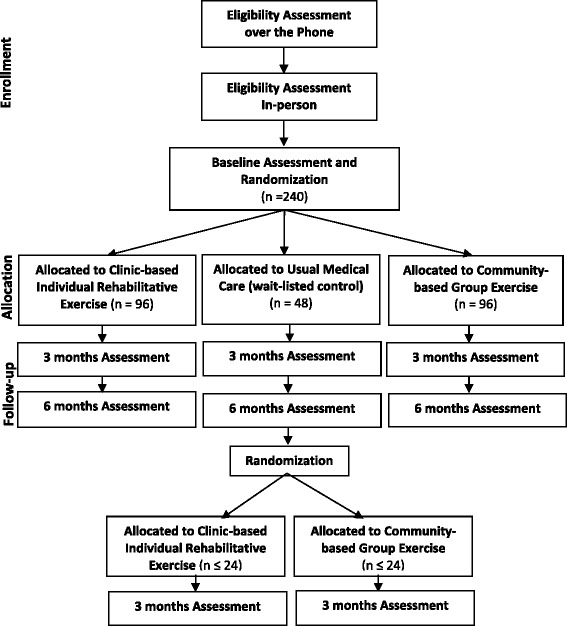
Study flow diagram

This comparative effectiveness study is designed as a three-group single-blind randomized clinical trial. Eligible subjects undergo baseline assessment and are randomized in a 2:2:1 allocation to one of the 3 groups: 1) clinic-based individual outpatient rehabilitative exercise; 2) community-based group exercise classes; or 3) usual medical care. The usual medical care group continues their usual care whereas the other two groups receive an exercise intervention for 12 weeks. Endpoint measures are assessed in-person at 3 and 6 months after randomization. Participants are also interviewed over the phone at 1.5 and 4.5 months after randomization to promote retention. After the 6 month follow-up, subjects in the usual medical care group are randomized to either clinic-based individual outpatient rehabilitation exercise or community-based group exercise and participate in a phone interview at 7.5 months and in-person assessment at 9 months after initial randomization. Figure 1 gives an overview of study design and procedures.

The design of this study is unbalanced with unequal number of subjects per group, meaning that the exercise groups have twice the number of subjects as the control group. The unbalanced design was chosen because larger functional recovery is expected in both exercise groups as compared to the usual care group, thus requiring larger sample size in the two exercise arms to detect smaller differences between them as compared to larger differences expected between either of the exercise arms and the usual care arm.

The study began in September 2014 and will continue for three years until August 2017. Ethics approval has been obtained from the University of Pittsburgh Institutional Review Board (PRO14080261). This protocol paper follows guidelines by the CONSORT Statement extensions for studies of non-pharmacological interventions [18] and pragmatic trials [19].

### Participants

The study enrolls adults older than 60 years of age from Allegheny County who underwent a unilateral TKR 2 to 4 months prior to study participation. Thus, subjects have healed from the surgical insult and knee pain, effusion, and motion are improved and no longer restrict more intense exercises. Participants also have to experience at least moderate functional limitation in daily activities to represent those with persistent limitations after TKR (minimum score of 9 points on the Western Ontario and McMaster Universities Osteoarthritis Index Physical Function Subscale -WOMAC-PF), speak English sufficient to understand study instructions, be willing to be randomized to one of the three treatment groups, and have a medical clearance to participate in the study. The study exclusion criteria are designed to ensure safety during exercise participation and minimize attrition: absolute or relative contraindications to exercise testing as established by the American College of Cardiology/American Heart Association [20, 21], uncontrolled cardiovascular disease or hypertension, history of neuromuscular disorder that affects lower extremity function, inability to walk 50 meters without an assistive device and to comfortably bear weight on the surgical knee, participation in structured exercise more than twice a week, terminal illness, plans to have another joint replacement or to relocate outside the immediate area during study period.

### Recruitment

The study uses several recruitment strategies. The primary strategy comprises of invitations sent to recent TKR patients directly from the knee surgeons who performed the procedure. Knee surgeons will recruit participants either during a regular follow-up visit or will send letters to their patients offering study participation. We anticipate participation in this recruitment strategy from 10 to 15 knee surgeons from several clinics, and who operate in different hospitals within Allegheny County. Additional recruitment strategies include direct mailings of postcards to local neighborhoods, study letters sent to participants of research registries, advertisements on local radio stations, newspapers, and other publications. People interested in the study are first screened by telephone and those deemed potentially eligible are scheduled for an in-person assessment to sign informed consent and re-confirm eligibility. If eligibility is confirmed during the in-person assessment, the participant undergoes a baseline assessment (Fig. 1).

### Randomization and masking

Patients are randomized using a 2:2:1 allocation ratio to receive one of the two exercise interventions as compared to usual medical care. The study coordinator performs the randomization through a web-based computer system at the end of baseline visit, thereby preserving allocation concealment. Single blinding is achieved by having the examiners who perform assessments masked to group assignment. To test for adequate masking, examiners will be asked to guess group assignment at the end of the study. While treatment assignment obviously cannot be masked to the participant, they are instructed not to discuss any aspects of the treatment with the examiners. Interventionists (physical therapists and exercise instructors) are masked to subjects’ performances on outcome measurements.

This study uses an adaptive randomization approach with minimal sufficient balance algorithm [22, 23] to minimize imbalances in important prognostic variables at baseline including gender, age, BMI, physical function, and knee range of motion. These measures have been selected due to their strong associations with the study outcomes of physical function and activity. Allocation is assigned based on the instantaneous imbalances instead of being generated as a fixed list prior to the beginning of the trial.

### Stakeholder participation

Our stakeholders include patients and providers, who have both been deeply engaged in developing this research study. Their input shaped the inclusion of the community-based exercise group, the decision for a control group wait-listed for exercise, and the selection of patient-centered outcomes. Patients also have approved recruitment materials relevant to older adults of all backgrounds, participated in a trial-run of the study procedures to ensure that materials are age-appropriate and research personnel are well trained. Stakeholders will continue to be engaged by their participation in an Advisory Panel comprised of: patients post TKR, physicians, physician assistants, physical therapists, as well as community and patient advocate organizations. The panel will oversee study implementation and provide their unique perspective in the interpretation of study results and dissemination.

### Interventions

The interventions used in this study are pragmatic and used in the manner they occur in the real-life clinical settings to help guide decision-making. The choice of interventions was based on input from patients, providers, our community stakeholders, and literature review.

#### Group 1 - Clinic-based individual outpatient rehabilitative exercise

The exercise program used in this group has been shown to be safe and feasible, and combines the best research evidence [14–17]. Subjects participate in 12 supervised sessions of exercise (60-minute each) followed by a home exercise program. The 12 sessions are supervised by a physical therapist during 3 months in the following schedule: 2 sessions per week during weeks 1–3; 1 session per week in weeks 4 to 7; and 1 session every 2 weeks for the last two visits. This gradual weaning is designed to allow enough time for the subjects to learn the exercises and increase adherence with the home exercise program. Participants are instructed to start home exercise after the 3rd week of the supervised program in a way that they exercise twice a week (either supervised exercise in the clinic or at home) during the 3-month intervention phase. Treatment sessions utilize a pragmatic approach and include: (1) warm-up with stretching of lower extremity muscles and range of motion exercises; (2) moderate to vigorous intensity strengthening exercises of the major lower extremity muscle groups (knee extensors, knee flexors, hip extensors and hip abductors); (3) moderate intensity aerobic training using a treadmill or exercise bicycle; (4) functional activities such as getting up from and sitting down in a chair, squatting, walking in place, kneeling, stair climbing and dancing; and (5) agility and balance exercises. All 5 components of the exercise program are used with each subject because patients with physical limitations post-TKR tend to be affected to varying degrees by these impairments. Exercises are initially at low intensity and are progressively increased to the target level, as long as subjects do not experience increased pain, effusion, or decreased range of knee motion. Individualization of exercise occurs in the selection of what exercises are emphasized in each component and the rate of exercise progression.

#### Group 2 - Community-based group exercise classes

Community-based exercise was selected as a comparator because stakeholders indicated that clinic-based physical therapy was expensive and sometimes difficult to get convenient appointments. Many patients indicated that they had tried community-based exercise as an alternative to structured physical therapy, in an effort to improve their surgical outcomes, and that they derived great benefit from it. We believe that community centers are a valuable – but under-utilized resource of the health care delivery system. Although some health insurance companies have begun to cover the costs of group exercise programs for their Medicare Advantage subscribers, few older adults take advantage of this benefit. Older patients with arthritis and other chronic conditions might benefit from community based exercise programs, which could be prescribed as a “treatment.” Yet, research is necessary to inform payers and policy makers about the potential value of community-based group exercise, which could represent a paradigm shift from the more conventional treatment approaches that involve clinic-based individualized exercise instruction.

Participants randomized to this group attend 60-minute group exercise classes for older adults at local community senior centers at the same frequency/duration as the clinic-based exercise group; 2 times per week during 3 months. The size of group exercise classes is variable but generally larger than 4 participants. The research participants attend classes along with non-research participants who are members of the community centers. The classes consist of a variety of exercises designed to increase general muscular strength, improve cardiovascular fitness, joint mobility, balance, and daily living skills. No specific body region is targeted with these exercise classes. Some of the exercises include: partial squats, leg and knee extension/flexion, elastic tubing or free weight for strength training of the upper arm and chest muscles, coordination drills with a gym ball such as bouncing, throwing and catching, and low-impact cardiovascular exercise using treadmill, bikes or aerobic series on the floor. The classes are taught by trained physical fitness instructors.

#### Group 3 - Usual medical care

In an effort to mirror current clinical conditions, no attempt is made to interfere with the care received from the doctor or independently sought by the participants in this group. While there is debate about what constitutes usual medical care at later stage post TKR, the definition of usual medical care was informed from extensive discussions with patients, physicians, rehabilitation clinicians, and a literature review which all indicate that at two months post TKR the majority of patients no longer undergo rehab despite suboptimal improvement [24–29]. Because the study only recruits subjects who had a TKR at least two months ago, it is unlikely that they are still undergoing rehabilitation. However, any subjects who are still participating in structured exercise 2 months after their TKR are excluded. Of note, in this study the usual care group serves as an effective wait list control group. After completing the 6-month control period, participants in this group are randomized to an exercise arm to equalize the potential benefits from study participation. This approach also intends to enhance compliance and address the ethical concern of asking volunteers to join an exercise research study and then asking them not to exercise.

### Baseline measures

At baseline, data are collected on demographics and biomedical characteristics, comorbidity, psychosocial factors, and impairments of the lower extremities. These data are used to characterize the sample and test potential predictors or modifiers of treatment response. Table 1 lists the measures collected at each time point.

**Table 1:** List of outcome measures collected at each time point

	Three intervention arms					Usual care only	
	BA	PA	IA	PA	IA	PA	IA
		1.5	3	4.5	6	7.5	9
Physical function							
Patient-reported function measured by the Western Ontario and McMaster Universities Osteoarthritis Index Physical Function (WOMAC-PF) subscale is the primary outcome [43, 44]. The WOMAC-PF consists of 17 items related to physical function. Each item is scored on a 5-point Likert-type Scale with descriptors from 0–4 (none, mild, moderate, severe, and extreme difficulty). Scores of each item are summed for a maximum total score on the WOMAC-PF of 68. Higher scores indicate worse functional limitations.	X		X		X		X
Performance-based function is measured by a battery of tests:	X		X		X		X
(1). Self-selected gait speed is assessed in m/sec while patients walk at their regular pace over 4 meters [32, 35].							
(2). Chair rise test times participants during 5 repetitions of rising to a full upright position and sitting back down in the chair without assistance. It uses a chair (18”height) without armrests [33, 34].							
(3). Single leg stance test records the time of balancing on one leg while keeping the hands on the hips. The test lasts up to 60 sec and is stopped if the swing leg touches the floor, support foot moves on the floor, or arms swing away from the hips [33, 34].							
(4). Stair ascend/descend test times participants while climbing up and down a set of 11 stairs (30 cm depth, 17 cm height) using a handrail on the preferred side [31].							
(5). Six min walk test assesses the distance covered while walking during 6 min on an unobstructed, rectangular circuit (marked in meters) [33, 34].							
(6). Sitting-rising test assesses the ability of participants to sit and rise from the floor [37].							
Results of these test are combined using a composite score formed with unit-weighted z scores of constituent tests to provide a more stable measure of the subjects’ underlying functional performance [45].							
Physical activity							
• Real-time physical activity is measured by the SenseWear Armband (SWA) (Body Media Inc., Pittsburgh PA). The SWA collects information from a tri-axial accelerometer, heat flux, skin temperature, and galvanic signal. The information is integrated and processed by software using proprietary algorithms to provide minute-by-minute estimates of light- and moderate-intensity physical activity. Participants wear the SWM on the back of the right arm during 24 hours/7 days (except during water activities) to obtain 5 complete days of data [46, 47].	X		X		X		X
• Self-reported physical activity is assessed using the Community Healthy Activities Model Program for Seniors questionnaire (CHAMPS). The CHAMPS is a valid instrument that provides information on the types of physical activities such as hobbies, work- and social-related activities, walking, swimming, and dancing [48, 49].	X		X		X		X
Demographics and biomedical characteristics							
Age, gender, race, education, BMI, self-rated health (excellent, good, fair, poor, or bad), discharge placement, number of prior rehabilitation sessions, surgical technique, and surgeon experience.	X						
Medication prescribed and over-the-counter used for pain.	X						
Comorbidity - assessed by the Cumulative Illness Rating Scale [50].	X						
Psychosocial factors							
• Fear-avoidance beliefs measured by the Tampa Scale for Kinesiophobia [51].	X		X		X		X
• Anxiety measured using the Beck Anxiety Index [52].	X		X		X		X
• Self-efficacy measured by the Arthritis Self Efficacy Scale [53].	X		X		X		X
• Depressive symptom assessed by the Center for Epidemiologic Studies Short Depression Scale [54].	X		X		X		X
• Pain coping measured by the Coping Strategy Questionnaire [55, 56].	X		X		X		X
Lower extremities impairments							
• Knee pain measured using an 11-point pain scale.	X		X		X		X
• Knee range of motion measured by a standard goniometer.	X		X		X		X
• Muscle strength of the knee extensors and hip abductor muscle groups using an isokinetic dynamometer (Biodex System 4 Pro, Shirley, NY) [57].	X		X		X		X
Safety and exploratory outcomes							
Adverse events - such as but not limited to changes in knee symptoms, falls, hospitalizations, and TKR on the other knee.		X	X	X	X	X	X
Attrition- defined as the number of patients dropping out of the study in each group.			X		X		X
Adherence to intervention- estimated by the proportion of sessions attended in each group and the proportion of patients missing each session.			X				X
Co-interventions- defined as additional treatment sought besides the ones prescribed by the study.		X	X	X	X	X	X

*BA* Baseline Assessment, *PA* Phone Assessments at 1.5, 4.5, and 7.5 months after randomization, *IA* In-person Assessments at 3, 6, and 9 months

### Outcome measures

The outcome measures are collected at baseline, 3 months, and 6 months follow-up. Patient stakeholders were consulted in the selection of outcome measures for this study. Thus, the outcomes of this study are patient-centered and reflect abilities that are important to patients post TKR. The primary outcome measure is physical function at the 3 month follow up assessed by a patient-reported survey, the Western Ontario and McMaster Universities Osteoarthritis Index Physical Function Subscale (WOMAC-PF) [30]. Secondary outcomes of physical function include a battery of performance-based tests easily performed in the clinical setting including self-selected gait speed, chair rise, single leg stance, stair climbing, six minute walk, and sitting-rising [31–37]. Additional secondary outcome includes physical activity measured by a portable monitor and a survey (Table 1). Safety and exploratory outcomes include the measures of harm assessed by adverse events and measures of study engagements including attrition, adherence to intervention, and participation in co-interventions, respectively.

### Data analysis

The primary hypothesis is that subjects in Groups 1 and 2 will demonstrate better physical function and physical activity as compared to Group 3 (usual medical care). Analysis for this hypothesis will use an intention-to-treat approach. Primary outcome for this analysis is the WOMAC-PF subscale at 3 months. This analysis will use contrasts from a linear mixed models analysis for 3 and 6 month function controlling for baseline function and the randomization covariates (age, gender, BMI, physical function, ROM). We will first explore the intervention by time interaction, and then proceed to a main effects model with only group and time. Our primary interest is the 3 month comparison between the clinic-based individual outpatient exercise and the community-based exercise groups. The linear mixed models allow maximization of the number of individuals used for the analyses, as a person can contribute information at both time points, or just at one time point. To test if the improvements in outcomes are sustained, we will use contrasts from the linear mixed model at 6 months. For the secondary outcomes, analyses are performed as described above, one for each measure. Among the performance based measures and the PA measures, Hochberg’s step-up procedure will be used to control the experiment-wise Type I error rate (α = 0.05) [38], which otherwise would be inflated due to the multiple endpoints.

Sample size and power calculations for primary analysis were based on the primary endpoint of WOMAC-PF subscale at 3 months. We propose to recruit 240 subjects (96 in each exercise arm and 48 in the usual care arm) to allow approximately 86 subjects in each exercise arm and 43 in the usual care arm available for a complete case analysis (assuming 10 % attrition at 3 months). With an alpha level of 0.05, 2 tails test, a sample size of 172 (*n* = 86 in each exercise group) will provide 81 % power to detect a difference of 3.3-point difference between the two exercise groups in WOMAC-PF (SD of 7.7) [17]. The sample size of 43 in the usual medical care group will provide 80 % power to detect a difference of 5.2-point difference in WOMAC-PF between the usual medical care group and any exercise group. Power analysis was conducted in NCSS/PASS (PASS 12 Power Analysis and Sample Size Software (2013). NCSS, LLC. Kaysville, Utah, USA, ncss.com/software/pass).

The secondary hypothesis is that a group of baseline biomedical and psychosocial measures will be associated with treatment response. For this analysis, each subject will be classified as a responder or non-responder based on a minimum change score of 20 % in both the WOMAC-PF and the composite score of functional performance at 3 months, thus yielding a binary outcome. Baseline variables will be summarized separately for responders and non-responders. Unadjusted odds ratios will be estimated using univariate logistic regression. To consolidate potential predictors, we will test for colinearity among baseline variables that are associated with response. Baseline measures associated with response at the *p* < 0.15 level in unadjusted models will be added to multivariable logistic regression models to assess predictors of treatment response. We will limit the number of predictors going into any one model to no more than one predictor per 10 responses (or 10 non-responses, whichever is less); if more variables are significant, the model will be limited to the most significant variables, after adjusting for those deemed a-priori to be clinically significant.

Power calculation for the secondary analysis is based on the binary outcome of 20 % change in physical function. Participants initially randomized to one of the exercise arms and those in the usual care group later randomized to the exercise arms will be included in the analysis for a total of approximately 200. If the expected response rate ranges between 50 % and 60 %, we would be able to detect an odds ratio of 2.2 to 2.4 with 80 % power assuming a binary predictor with 50 % split in the sample.

For the exploratory aim we will calculate dropout rates as proportions of subjects randomized and as a cumulative probability of remaining in the study using survival analysis techniques, such as the product-limit estimator. These statistics can be estimated at various times following randomization and take into account when dropouts occur. Descriptive statistics will be used for reporting and evaluating implementation of the exercise protocols including the proportion in attendance for each session and the average number of sessions attended by group. To assess the impact of non-adherence, we propose to explore using instrumental variable (IV) methodology to estimate the efficacy for our interventions in the presence of non-adherence [39, 40]. We propose to use the two-stage IV methods which can be easily implemented using simple linear structural models for the effect of sessions attended on the primary outcome of function. We will also calculate the 6-month incidence (and 95 % CI) of individual adverse events by organ system and relatedness to the study for each group. We will estimate the incidence of adverse events with specific focus on those deemed definitely, probably, or possibly related to interventions. For adverse events, clinical judgments will be considered more important than statistical testing.

We also propose sub-group analyses to explore heterogeneity of treatment effects using several potential moderators of treatment response measured prior to randomization that may either potentiate or attenuate the effects of our intervention (e.g., patient gender, age, BMI, range of knee motion). These are the same prognostic variables used for the adaptive randomization in the study. We will examine interactions between the treatment and modifier being considered. Even if the interaction is not statistically significant, we would estimate the treatment effects stratified by age along with the 95 % confidence intervals to look for consistency of treatment effects.

### Improving adherence and dealing with missing data

To prevent missing follow-up assessments, all participants will be reimbursed for testing and parking expenses and the research coordinator will interview them regularly by telephone to promote engagement in the study. We will also attempt to collect follow-up data for as many of these subjects as possible. For example, if a participant cannot come for an in-person follow-up assessment, the participant is offered to complete the surveys at home and mail them back to the study staff. Of note, subjects in the three study arms will participate in exercise programs and will likely have positive expectations of treatment benefit, which should facilitate study retention and decrease missing data. Despite attempts to improve adherence, some missing data are expected. To deal with missing data, baseline characteristics between patients with and without the assessment at 3 and 6 months will be compared to assess potential biases in the complete case analysis. We will also try to obtain reasons for study drop out to assess the missing data mechanism (missing completely at random, missing at random, non-ignorable missingness). We will use several missing data methods for imputing data and re-analyze using intention to treat (as randomized) to assess the impact of missing data on our conclusions as recommended [41].

### Reproducibility of study procedures

We will develop a Manual of Operations and Procedures (MOP) to standardize all procedures and staff training in areas such as patient recruitment, measurement, assessment, data entry, management, analysis, and security. The MOP will also delineate the monitoring plans to assure patient protection and data integrity, thus facilitating consistency in protocol implementation and data collection. Reproducibility of testing procedures will be attained by conducting regular training workshops with the testers during which all the examination procedures will be reviewed and practiced. The training will be repeated yearly and involves role playing and observation of interviews carried out by the tester by experienced interviewers. Reproducibility of interventions will be maintained by regular meetings with the physical therapists and exercise instructors to review the research protocols to ensure treatment consistency.

## Discussion

TKR is a highly prevalent and expensive surgical procedure. While TKR decreases pain in most patients, it does not resolve many of the substantial functional limitations associated with chronic knee arthritis that existed for a long time prior to the surgery. Exercise programs could improve these long-term limitations if implemented at a later stage post TKR when patients can tolerate sufficient exercise intensity. Yet, there is not enough information to guide which type of exercise works best for which patients at a later stage post TKR. As a consequence, the majority of patients ends up receiving sub-standard care and are prematurely discharged from rehabilitation before the exercises can be intensified to enhance surgical outcome. This is an area of research that has been overlooked despite effect on patients, clinicians and health care payers. This study will provide evidence to inform the choice of exercise programs at the later stages after surgery to guide decisions on prevention of morbidity and ways to maximize the benefits of TKR. It is also unknown which patients benefit from exercise at a later stage post-TKR and this study will identify predictors of functional recovery and determine which treatment works best for outcomes relevant to patients. Last, the study will investigate the potential harms of comparators.

This comparative effectiveness study is unique because it is designed with considerable input from stakeholders, particularly patients. It combines patient-centered research questions with rigorous research methods that minimize bias and balance internal and external validity. In an attempt to increase applicability of interventions in clinical settings we have selected some domains of trial design that are equally pragmatic/explanatory whereas others are rather pragmatic. These domains are discussed based on the PRECIS-2 tool [42].

The eligibility domain is rather pragmatic as it promotes inclusion of individuals with TKR who are the real candidates for late stage exercise programs. Inclusion criteria are broad and offer no restrictions related to older age or range of comorbidities. Enrolling older adults who have multiple co-morbidities such as obesity, diabetes, high blood pressure and arthritis of other joints will improve applicability of study results. Moreover, the exclusion criteria are narrow and mainly based on safety. The safety criteria mimic real world as patients with contraindications to exercise would not be referred to participation in intense exercise programs in the community or in non-specialized rehabilitation settings, and those who cannot comfortably bear weight on the surgical knee are not indicated for participation in exercise programs until assessment to rule out causes such as infection or loosening of prosthesis. While the exclusion of individuals not likely to participate in follow-up testing (i.e., terminal illness, TKR planned for the other knee, or plan to move to other location) compromises pragmatism, there is no exclusion based on social disadvantage, mental health problems, or poor motivation. Additionally, as exercise compliance is always an issue in clinical settings, to increase applicability we excluded those known to be highly compliant with exercise (i.e., participate in structured exercise more than twice a week). The exclusion of participants with history of neuromuscular disorder had the purpose to prevent confounding but also eliminates participants not likely to respond to treatment response; i.e., not aligned with pragmatism.

In the domain of recruitment, this trial is rather pragmatic as the primary recruitment source is the knee surgeons and represents the same setting intended to apply the study results. While we anticipate that this method will provide the vast majority of the study sample, we also plan to use recruitment strategies such as research registries and public announcement if needed to speed up recruitment, which slightly reduces pragmatism. In terms of setting and organization of interventions, the community-based group exercise makes no modification in the exercise classes delivered to older adults in community centers. In the usual medical care group, to mirror clinical conditions we are careful not to interfere with the care received. The clinic-based individual rehabilitative exercise provides care with resources that are available in the outpatient setting. However, we acknowledge that some outpatient clinics may not follow the best current evidence-based exercise by neither regulating exercise delivery nor progression, which compromises pragmatism. In the analysis domain, the trial is pragmatic as we will use intention to treat with all available physical function and physical activity data.

Last, we recognize that the follow-ups in the study are more frequent and longer than in the real-world clinical setting, and also that some outcomes measures are not widely used in clinical practice. For example, the WOMAC and real-time physical activity monitoring are costly and seldom used in the clinics, and the data processing of physical activity monitoring is cumbersome for clinical use. However, these measures are widely used in clinical research and are genuinely important to patients; physical function and activity are two of the most patient-centered outcomes reported to us by patients post TKR. Thus, in the continuum of explanatory or pragmatic trial designs, the overall balance of study domains places this study towards the pragmatic side and will allow more direct application of the information and help decision makers choose whether to implement the interventions tested in the trial.

## Abbreviations

MOP: Manual of operations and procedures
PCORI: Patient-Centered Outcomes Research Institute
TKR: Total knee replacement
IV: Instrumental variable
WOMAC-PF: Western Ontario and McMaster Universities osteoarthritis index physical function subscale
